# Superior Ophthalmic Vein Thrombosis Presenting as Isolated Headache: A Case Report

**DOI:** 10.1159/crn/abyag020

**Published:** 2026-10-10

**Authors:** Vincenzo Laterza, Alfredo De Liso, Francesco Macchione, Alessandra Bavaro, Marco Rossi, Giuseppe Rinaldi

**Affiliations:** Department of Neuroscience, “Di Venere Hospital”, Bari, Italy

**Keywords:** Superior ophthalmic vein thrombosis, Orbital pain, Headache, Antiphospholipid syndrome, Case report, Anticoagulation

## Abstract

**Introduction:**

Superior ophthalmic vein thrombosis (SOVT) is a rare condition that can present with varied symptoms, including headache and orbital pain. Its diagnosis requires advanced imaging techniques, including CT angiography and MRI. This case report describes a patient presenting with headache and orbital pain attributed to superior ophthalmic vein dilation and thrombosis.

**Case Presentation:**

An 81-year-old female patient presented with headache, orbital pain, periorbital edema, and mild palpebral ptosis. Initial investigations included a brain CT scan and CT angiography, which revealed tortuous and dilated superior ophthalmic veins with massive thrombosis of the left superior ophthalmic vein. Brain MRI and MR angiography were also performed. Other examinations included ophthalmological evaluation, fluorescein angiography, and blood tests. The patient’s symptoms, including headache and orbital pain, were attributed to the confirmed dilation and massive thrombosis of the left superior ophthalmic vein. CT angiography initially suggested a carotid-cavernous fistula, which was subsequently excluded by brain MRI and MR angiography. Fluorescein angiography revealed severe myopic chorioretinopathy with peripapillary atrophy and disc hyperfluorescence consistent with venous congestion in the left eye. Thrombophilia screening identified elevated anticardiolipin IgM antibodies, raising suspicion for antiphospholipid syndrome (APS). Anticoagulant therapy was initiated with enoxaparin and subsequently transitioned to warfarin, in accordance with current guidelines that favor vitamin K antagonists over direct oral anticoagulants for APS-associated thrombosis.

**Conclusion:**

This case highlights the importance of considering SOVT in the differential diagnosis of headache accompanied by orbital symptoms.

## Introduction

Headache is a common neurological symptom with a wide range of etiologies, from benign primary headaches to those indicative of serious underlying conditions. Orbital pain, periorbital edema, and palpebral ptosis, when accompanying headache, can suggest an orbital or retro-orbital pathology [1]. Superior ophthalmic vein thrombosis (SOVT) is a rare [2, 3] but potentially vision-threatening and life-threatening condition that can manifest with such symptoms [4]. SOVT can be primary or secondary to various causes, including inflammation, infection, trauma, hypercoagulable states, or vascular malformations such as carotid-cavernous fistulas [4]. The diagnosis of SOVT often relies on advanced imaging techniques, such as CT angiography, MR angiography, or conventional angiography [3]. Given its rarity and the potential for severe complications, prompt recognition and appropriate management are crucial [4]. This case report aims to describe a patient presenting with headache and orbital pain, ultimately diagnosed with left superior ophthalmic vein dilation and thrombosis, and to discuss the relevant clinical and diagnostic considerations. This case report was prepared in accordance with the Case Report (CARE) guidelines, and the completed CARE checklist is provided as online supplementary material (for all online suppl. material, see https://doi.org/10.1159/crn/abyag020).

## Case Report

An 81-year-old female patient presented to the emergency department with a 7-day history of progressively worsening headache and left orbital pain, accompanied by periorbital edema and mild palpebral ptosis. Her medical history included dyslipidemia and hypertension, both under treatment, severe myopia in the right eye complicated by cataract in both eyes, and an anxiety disorder.

The patient reported no personal or family history of thromboembolic events, including deep vein thrombosis, pulmonary embolism, or ischemic stroke. No history of pregnancy morbidity was reported.

Initial evaluation at the emergency department included a brain CT scan and CT angiography. The latter revealed tortuous and dilated superior ophthalmic veins, with evidence of massive thrombosis of the left superior ophthalmic vein (Fig. 1). The findings suggested a possible underlying vascular malformation, such as a carotid-cavernous fistula, though this was not definitively documented with the acquired vascular phase. Neurosurgical and interventional neuroradiology consultations were obtained, which excluded an urgent need for angiography but recommended further imaging with MRI and MR angiography, and eventual cerebral angiography in elective settings. Upon admission, neurological examination revealed the patient to be awake, conscious, and oriented. Cranial nerves were intact, with normal extraocular movements, minimal ptosis in the left eye, and no meningeal signs. Motor and sensory functions were normal in all four limbs, with normal tone and trophism. The Romberg test was negative, and cerebellar tests were correctly performed. Gait was autonomous with no clear pathological features, though tandem gait was slightly difficult. During hospitalization, the patient’s clinical condition remained stable. Relevant laboratory findings included elevated NT-ProBNP (227.00 pg/mL, reference range up to 125.00 pg/mL), elevated creatine kinase (168.00 U/L, reference range 34.00–145.00 U/L), elevated lactate dehydrogenase (344.00 U/L, reference range 120.00–246.00 U/L), elevated Fibrinogen (423 mg/dL, reference range 150–400 mg/dL). Blood tests, including thrombophilia screening, neoplastic markers, an autoimmune panel, and thyroid function tests, were performed. Laboratory workup revealed mild hyperhomocysteinemia in the setting of compound MTHFR mutation (heterozygous C677T and A1298C polymorphisms), low levels of folic acid. Anticardiolipin antibody testing showed elevated IgM titers (31.2 U/mL; reference range 0–10 U/mL) with negative IgG. Inflammatory markers were normal. Infectious investigations were also negative. Coagulation testing showed a normal prothrombin time (INR 0.93) and activated partial thromboplastin time (ratio 0.80), with a D-dimer of 0.658 mg/L FEU.

**Fig. 1.**
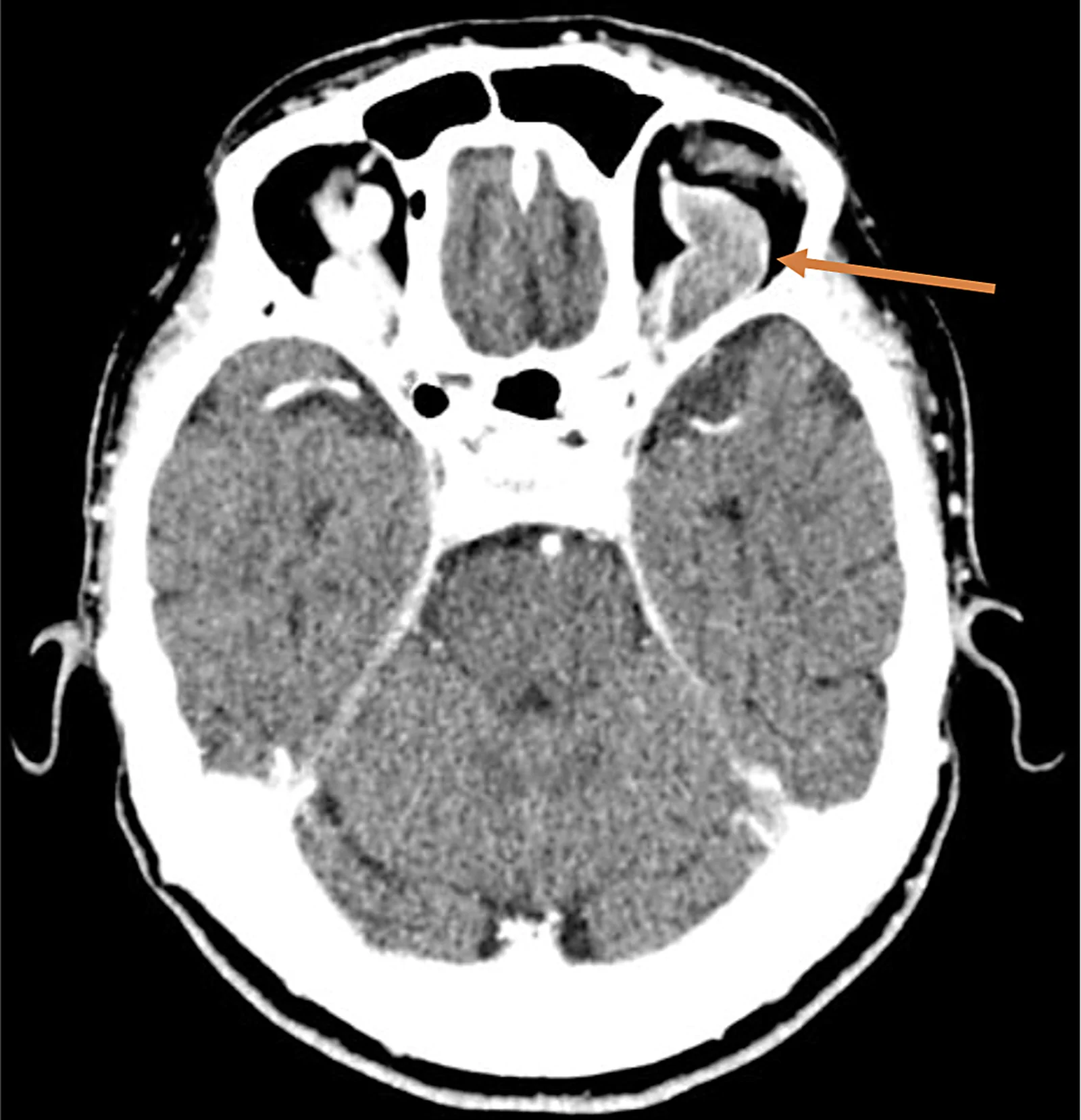
Axial CT angiography demonstrating a filling defect within the left ophthalmic vein, consistent with thrombosis.

Given the elevated anticardiolipin IgM titers identified during thrombophilia screening, antiphospholipid syndrome (APS) was suspected. An ophthalmological evaluation and fluorescein angiography were performed.

Ophthalmological examination showed best-corrected visual acuity of 3/10 in the right eye and 1/10 in the left eye. Pupillary responses were normal bilaterally, with no relative afferent pupillary defect. Ocular motility was intact in all directions of gaze, with no diplopia or chemosis. Mild proptosis of the left globe was present. Intraocular pressure was 13 mm Hg in the right eye and 15 mm Hg in the left eye at the specialist evaluation; at initial presentation in the emergency department a transiently elevated value of 22 mm Hg had been recorded in the left eye, in the context of a suspected subacute angle-closure attack treated with intravenous mannitol. Fundoscopic examination showed sharp optic-disc margins, mildly tortuous retinal vessels, and no retinal hemorrhages or cotton-wool spots. Color vision was not formally assessed.

Fluorescein angiography of the left eye showed severe myopic chorioretinopathy with extensive peripapillary atrophy, and the optic disc showed hyperfluorescence in the intermediate and late phases along its circumference, consistent with venous congestion, with changes in the retinal pigment epithelium at the posterior pole. The MRI and MR angiography confirm the presence of dilation of the left ophthalmic vein due to thrombosis (Fig. 2–3). Brain MRI showed no acute ischemic lesions, intracranial hemorrhage, or parenchymal signal abnormalities. Anticoagulant therapy with enoxaparin 4000 I.U. twice daily was started. The patient was discharged in stable clinical condition with a diagnosis of left ophthalmic vein thrombosis. Discharge medications included rosuvastatin 5 mg, ramipril 10 mg, and enoxaparin 4000 IU twice daily, initiated as bridging therapy pending transition to warfarin in the context of suspected APS. Oral anticoagulation with warfarin was subsequently initiated and overlapped with enoxaparin until a therapeutic INR was achieved. Following discharge, the patient reported mild left-sided periorbital pain, managed with paracetamol 1,000 mg as needed. Follow-up instructions included a control brain CT, which confirmed the ectatic and tortuous appearance of the bilateral superior ophthalmic veins. Compared to the previous scan, it demonstrated an eccentric filling defect at the proximal segment of both veins, consistent with thrombosis and more pronounced on the left side.

**Fig. 2.**
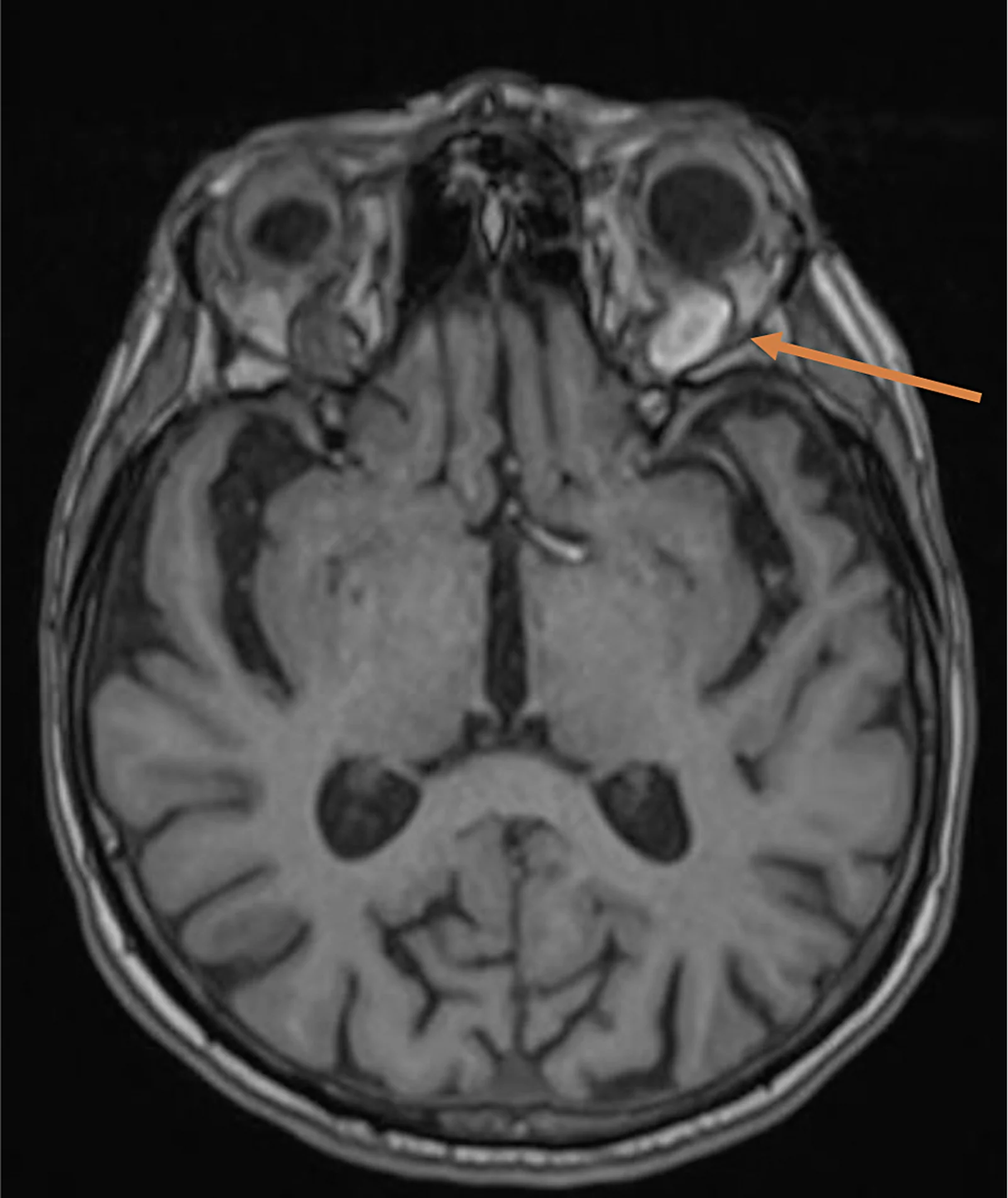
Axial FLAIR brain MRI at the orbital level showing findings consistent with left ophthalmic vein thrombosis.

**Fig. 3.**
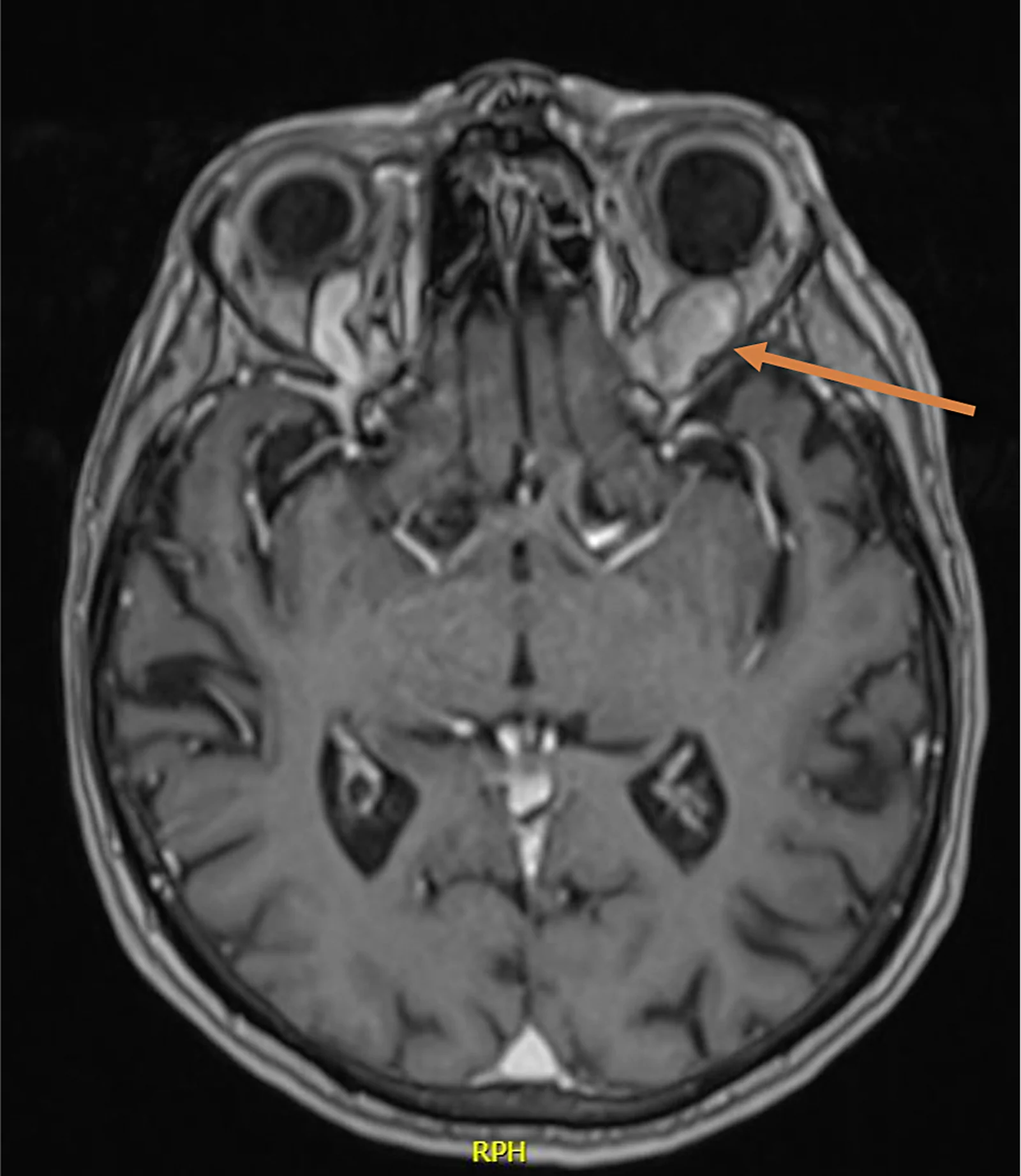
MR venography (MRV) demonstrating absence of flow in the left ophthalmic vein, consistent with thrombosis.

## Discussion

This case illustrates a rare presentation of headache and orbital pain secondary to superior ophthalmic vein dilation and thrombosis [2, 3]. To date, to the best of our knowledge and based on a PubMed literature search, only two cases of SOVT secondary to APS have been reported [5, 6]. The initial suspicion of a carotid-cavernous fistula, though later excluded, highlights the importance of a thorough diagnostic workup in such cases as these conditions can present with overlapping orbital signs and symptoms due to similarly altered venous drainage patterns [4]. CT angiography proved essential in the initial assessment, while MRI and MR angiography provided definitive confirmation of the thrombosis and allowed exclusion of other mimicking disorders, including cavernous sinus thrombosis and sino-orbital infection The differential diagnosis also included orbital inflammatory disease (orbital pseudotumour) and compressive orbital lesions; both were considered unlikely in the absence of an orbital mass and given the imaging demonstration of an intraluminal venous filling defect.

The carotid-cavernous fistula was excluded based on MRI and MR angiography, which showed no arteriovenous communication or arterialization of the superior ophthalmic vein. Digital subtraction angiography was not performed as non-invasive imaging provided a clear picture consistent with venous thrombosis without high-flow features. Lumbar puncture was not carried out as there were no clinical signs of raised intracranial pressure: the patient had no progressive visual loss, no sixth nerve palsy, and neuroimaging was unremarkable for signs of intracranial hypertension [7]. The thrombophilic workup performed during hospitalization identified several potential contributing factors. Mild hyperhomocysteinemia in the context of compound MTHFR mutation (heterozygous C677T and A1298c polymorphisms) and low folic acid levels represent a recognized, albeit modest, prothrombotic risk factor. More significantly, elevated anticardiolipin IgM titers raised the suspicion of APS, a systemic autoimmune condition strongly associated with both arterial and venous thrombosis. It should be noted, however, that a formal diagnosis of APS requires confirmation of antibody positivity on two separate occasions at least 12 weeks apart [8]. At 12-week follow-up, repeat testing confirmed persistent anticardiolipin IgM positivity, fulfilling the laboratory criteria for a definite diagnosis of APS. It is worth noting that our patient's age represents an unusual feature in the context of APS. The syndrome typically affects young to middle-aged women, with most thrombotic events occurring between the third and fourth decade of life. An 81-year-old patient with APS-associated SOVT is therefore a highly atypical presentation. This highlights a potential risk of underdiagnosis in the elderly, where thrombotic events are often attributed to conventional cardiovascular risk factors – such as hypertension and dyslipidemia, both present in our patient – without pursuing a dedicated thrombophilic workup. Our patient carried a compound MTHFR mutation with mild hyperhomocysteinemia, alongside elevated anticardiolipin IgM antibodies, suggesting a combination of inherited and acquired thrombophilic factors. Although the MTHFR mutation alone is a relatively weak prothrombotic risk factor, its co-occurrence with APS may have tipped the balance toward thrombosis in this unusual location. This serves as a reminder that in patients with venous thrombosis at atypical sites, a thorough thrombophilic workup should always be pursued. In accordance with current guidelines, which favor warfarin over direct oral anticoagulants in APS-associated thrombosis due to the higher risk of recurrence with DOACs in this setting, anticoagulation was transitioned from enoxaparin to warfarin following discharge.

The mild palpebral ptosis in our patient was most likely related to venous congestion and orbital edema rather than levator dysfunction or cranial nerve III involvement, given the complete preservation of ocular motility and the absence of any other oculomotor deficit. The disc hyperfluorescence seen on fluorescein angiography, together with sharp optic-disc margins on fundoscopy, is best interpreted as a sign of orbital venous congestion rather than true papilledema; accordingly, no clinical or radiological signs of raised intracranial pressure were present in our patient. The prompt initiation of anticoagulant therapy is crucial in managing SOVT to prevent thrombus propagation and reduce the risk of visual impairment or other neurological complications [4]. This case underscores the need for a high index of suspicion for SOVT in patients presenting with headache and orbital symptoms, even in the absence of overt signs of orbital inflammation or infection. Multidisciplinary management involving neurologists, ophthalmologists, and interventional neuroradiologists is essential to ensure optimal diagnostic and therapeutic decision-making.

## Conclusions

Superior ophthalmic vein dilation and thrombosis should be considered in the differential diagnosis of headache accompanied by orbital pain, periorbital edema, and ptosis. Early and accurate diagnosis, guided by appropriate imaging studies, is critical for effective management and prevention of adverse outcomes. This case emphasizes the importance of a comprehensive clinical and laboratory evaluation to identify predisposing factors and to guide therapeutic interventions. In particular, APS should always be ruled out in patients with SOVT of uncertain cause as its recognition directly influences treatment choices, notably the preference for warfarin over newer oral anticoagulants. Further research is needed to better understand the pathophysiology of SOVT and its association with systemic conditions.

## Statement of Ethics

This study was performed in accordance with the Declaration of Helsinki. Ethical approval is not required for individual case reports at our institution in accordance with local guidelines. Written informed consent was obtained from the patient for publication of the details of their medical case and any accompanying images.

## Conflict of Interest Statement

The authors have no conflicts of interest to declare.

## Funding Sources

This research received no specific grant from any funding agency in the public, commercial, or not-for-profit sectors.

## Author Contributions

V.L.: conceptualized the study, curated the data, and wrote the original draft. A.D.L. and F.M. contributed to clinical management and data curation. A.B. and M.R. performed the ophthalmological evaluation, curated the data, and reviewed and edited the manuscript. G.R. supervised the project and reviewed the manuscript.

## Data Availability

All data generated or analyzed during this study are included in this article. Further inquiries can be directed to the corresponding author.
